# Genome Sequence of *Brevibacillus laterosporus* UNISS 18, a Pathogen of Mosquitoes and Flies

**DOI:** 10.1128/genomeA.00419-17

**Published:** 2017-05-25

**Authors:** Salvatore Camiolo, Andrea Porceddu, Luca Ruiu

**Affiliations:** Dipartimento di Agraria, University of Sassari, Sassari, Italy

## Abstract

The entomopathogenic properties of *Brevibacillus laterosporus* UNISS 18 against insects are well documented. Here, we report the whole-genome sequence of this strain, which revealed the presence of several insecticide action-related genes. The deriving genetic information will help to elucidate the mechanisms underlying strain specificity and virulence against diverse target pests.

## GENOME ANNOUNCEMENT

*Brevibacillus laterosporus* Laubach is a Gram-positive, rod-shaped, aerobic (facultative anaerobic), and ubiquitous bacterial species. It belongs to the family *Paenibacillaceae* and is characterized by swollen sporangia containing a typical canoe-shaped parasporal body (CSPB) laterally associated with the spore (1). The biological properties of this bacterium range from the biopesticidal potential of certain strains against diverse target insects and phytopathogens (bacteria and fungi) to a broad-spectrum antimicrobial activity with pharmaceutical applications (2).

*B. laterosporus* UNISS 18 (=NCIMB 41419) was originally isolated from soil and its insecticidal action, relying on a toxin-mediated process taking place in the insect midgut after spore ingestion, has been well-documented against mosquitoes and house flies (3, 4). Sequencing, assembly and annotation of the *B. laterospours* UNISS 18 genome provides valuable information for the identification of new putative insect toxins and virulence factors.

Genomic DNA was isolated employing the DNeasy blood and tissue kit (QIAGEN GmbH, Hilden, Germany). DNA was quantified using the Qubit 2.0 Fluorometer (Life Technologies, Carlsbad, CA, USA) and supplied to the next-generation sequencing facilities of Porto Conte Ricerche Srl (Tramariglio, Alghero, Italy). Libraries were generated according to the Nextera XT DNA sample preparation protocol (Illumina, San Diego, CA, USA). Normalized sample libraries were pooled and subjected to the cluster generation step using the cBOT cluster generation station, according to the Illumina TruSeq paired-end cluster kit protocol. DNA sequencing was performed with the Illumina HiScanSQ sequencer, using the paired-end approach. The produced 93 bp reads (9,037,662) were quality-filtered using the software application NGS QC toolkit (5). The resulting data set was used to assemble the UNISS 18 genome by using the software SPAdes (6). After removing assembled sequences shorter than 500 bp, 64 scaffolds were obtained accounting for 5,333,788 bp and with a 41.1% G+C content. The genome was annotated using the RAST web server (7), which allowed the identification of 4,874 protein coding genes together with 15 rRNA and 57 tRNA genes. Finally, the tool Blast2GO (8) was used to investigate the functional annotation (biological function, cellular component, and cellular process) of the predicted transcripts, leading to 3,393 genes with valid ontology.

In order to gain a deeper insight into the *B. laterospours* UNISS 18 produced assembly, the quality-filtered reads were aligned to the *B. laterospours* LMG 15441 reference genome using Altools (9). This analysis led to the detection of 44,122 single nucleotide polymorphisms (SNPs) and 1,291 indels with such variations being possibly associated with the strain specific virulence. In this regard, polyketide synthases (PKS), nonribosomal peptide synthases (NRPS), and genes encoding putative toxins (10) proved to be affected by a relevant number of the identified polymorphisms.

### Accession number(s).

This whole-genome shotgun project has been deposited at DDBJ/ENA/GenBank under the accession no. MBFH00000000. The version described in this paper is version MBFH01000000.
